# Effect of low-dose rituximab treatment on autoimmune nodopathy with anti-contactin 1 antibody

**DOI:** 10.3389/fimmu.2022.939062

**Published:** 2022-07-26

**Authors:** Ying Hou, Chao Zhang, Xiaolin Yu, Wenqing Wang, Dong Zhang, Yunfei Bai, Chuanzhu Yan, Lin Ma, Anning Li, Jian Ji, Lili Cao, Qinzhou Wang

**Affiliations:** ^1^ Research Institute of Neuromuscular and Neurodegenerative Diseases and Department of Neurology, Qilu Hospital, Cheeloo College of Medicine, Shandong University, Jinan, China; ^2^ Department of Neurosurgery, Qilu Hospital, Cheeloo College of Medicine, Shandong University, Jinan, China; ^3^ Department of Geriatric Medicine, Qilu Hospital, Cheeloo College of Medicine, Shandong University, Jinan, China; ^4^ Key Laboratory of Cardiovascular Proteomics of Shandong Province, Qilu Hospital, Cheeloo College of Medicine, Shandong University, Jinan, China; ^5^ Department of Central Laboratory and Mitochondrial Medicine Laboratory, Qilu Hospital (Qingdao), Cheeloo College of Medicine, Shandong University, Qingdao, China; ^6^ Brain Science Research Institute, Shandong University, Jinan, China; ^7^ Department of Radiology, Qilu Hospital, Cheeloo College of Medicine, Shandong University, Jinan, China; ^8^ Department of Clinical Laboratory, Qilu Hospital, Cheeloo College of Medicine, Shandong University, Jinan, China; ^9^ Department of Neurology, Qilu Hospital, Cheeloo College of Medicine, Shandong University, Jinan, China

**Keywords:** anti-CNTN1 antibodies, low-dose rituximab, treatment outcome, autoimmune nodopathy, antibody titer

## Abstract

**Background:**

Autoimmune nodopathy with anti-contactin-1 (CNTN1) responds well to rituximab instead of traditional therapies. Although a low-dose rituximab regimen was administered to patients with other autoimmune diseases, such as myasthenia gravis and neuromyelitis optica spectrum disorders, and satisfactory outcomes were obtained, this low-dose rituximab regimen has not been trialed in anti-CNTN1-positive patients.

**Methods:**

Anti–CNTN1 nodopathy patients were enrolled in this prospective, open-label, self-controlled pilot study. A cell-based assay was used to detect anti-CNTN1 antibodies and their subclasses in both serum and cerebrospinal fluid. Clinical features were evaluated at baseline, 2 days, 14 days, and 6 months after single low-dose rituximab treatment (600 mg). The titers of the subclasses of anti-CNTN1 antibody and peripheral B cells were also evaluated at baseline, 2 days, and 6 months after the rituximab regimen.

**Results:**

Two patients with anti–CNTN1 antibodies were enrolled. Both patients had neurological symptoms including muscle weakness, tremor, sensory ataxia, numbness and mild nephrotic symptoms. In the field of neurological symptoms, sensory ataxia markedly improved, and the titer of anti-CNTN1 antibody as well as CD19+ B cells decreased only two days following low-dose rituximab treatment. Other neurological symptoms improved within two weeks of rituximab treatment. At the 6-month follow-up, all neurological symptoms steadily improved with steroid reduction, and both the anti-CNTN1 antibody titer and CD19+ B cells steadily decreased. No adverse events were observed after this single low-dose rituximab treatment.

**Conclusions:**

We confirmed the clinical efficacy of low-dose rituximab by B cell depletion in autoimmune nodopathy with anti-CNTN1 antibody. This rapid and long-lasting response suggests that low-dose rituximab is a promising option for anti-CNTN1 nodopathy.

## Introduction

Autoimmune nodopathies were defined as antibodies against nodal-paranodal cell-adhesion molecules such as neurofascin155 (NF155), contactin-1 (CNTN1), contactin-associated protein 1 (Caspr1), and neurofascin140/neurofascin186 (NF140/186) (1). Unlike typical chronic inflammatory demyelinating polyneuropathy (CIDP), patients with these antibodies often have specific clinicopathological features such as tremor and sensory ataxia, and show a poor response to traditional therapies such as intravenous immunoglobulin (IVIg) and prednisone treatment (2, 3). Therefore, autoimmune nodopathy is now considered a separate entity rather than a subgroup of CIDP based on the latest criteria (1).

The unique pathological findings of anti-CNTN1 nodopathy that the detachment of myelin terminal loops from the axolemma at the paranode was quite different from that in conventional CIDP as the phagocytosis of myelin by macrophages. Thus, patients with anti-CNTN1 nodopathy have distinct immunotherapy (4). The most common isotype of the anti-CNTN1 antibody is IgG4. Similar to other IgG4 autoimmune diseases such as myasthenia gravis with antibodies to muscle-specific kinase (MuSK-MG), anti-CNTN1 autoimmune nodopathy generally responds well to rituximab, a monoclonal antibody against B lymphocyte membrane protein CD20 (5, 6). Specifically, improvements in neurological symptoms including gait, tremor, muscle strength, and a decrease in antibody titer can be seen, especially in those who are resistant to IVIg and prednisone (7–9). However, the high cost of the common-dose rituximab regimen such as 375 mg/m^2^/week over 4 weeks, and some adverse events have restricted its widespread use in China.

Indeed, many patients with autoimmune diseases, including anti-NF155 autoimmune nodopathies, neuromyelitis optica spectrum disorder (NMOSD), MG, and Isaacs syndrome, received low-dose rituximab with a total dose of 600 mg, separated as 100 mg on the first day and 500 mg on the second day (10–13). Although this low-dose regimen could also lead to improvement of clinical manifestations, a decrease in antibody titers, and a reduction of the average steroid dosage by B cell elimination and subsequent B cell repopulation (10–12), the research of this low-dose rituximab regimen in anti-CNTN1 nodopathy is limited. Herein, we report the effects of this low-dose rituximab regimen in patients with anti-CNTN1 antibodies.

## Materials and methods

### Participants and study design

We included all patients seen in the Department of Neurology at Qilu Hospital who met the definite electrodiagnostic criteria for CIDP based on the European Federation of Neurological Societies/Peripheral Nerve Society Guideline criteria (2010) with antibodies against CNTN1. They were diagnosed by two experienced neurologists (YH and Q-zW) (14).

Low-dose of rituximab (600 mg over two consecutive days, 100 mg on day 1 and 500 mg on day 2) was administered to these patients. The dosage of steroids was adjusted according to the clinical status during the subsequent treatment period. Clinical evaluation included the Hughes disability scale, modified Rankin score (mRS), and overall disability sum score (ODSS). Treatment responses were defined in terms of △Hughes (the scale value after treatment minus that before treatment) as follows: △Hughes < 0, effective; △Hughes = 0, with subjective or objective improvement, partially effective; and △Hughes ≥0, without any improvement, ineffective. Clinical features were evaluated at baseline and 2 days, 14 days and 6 months after rituximab treatment. Peripheral blood was also collected at baseline (time of initiation of rituximab) and post-treatment (2 days, 14 days and 6 months after rituximab infusion).

### Antibody detection

In both serum and cerebrospinal fluid (CSF), anti-CNTN1 autoantibody and its subclasses were detected using a cell-based assay method. Specifically, the human CNTN1 coding sequence (NM_001843.4) was subcloned into the pcDNA3.1 plasmid, and transfected into HEK293T cells. After 48 h, cells were fixed with cold acetone. Fixed cells were incubated with patients’ serum diluted with PBS or CSF for 1 h, then incubated with corresponding FITC labeled secondary antibodies (FITC-goat anti-human IgG Fcγ antibody (109-095-170, Jackson ImmunoResearch, PA, USA) for IgG detection; FITC-goat anti-human IgG1 (F0767, Sigma-Aldrich, MO, USA), IgG2 (F4516), IgG3 (F4641), and IgG4 (F9890) antibodies for IgG subclass detection) at 1:200 dilution for 30 min. Autoantibody reactivity was examined using fluorescence microscope (Leica).

To confirm that the anti-CNTN1 antibody was located in the paranodal region, an immunofluorescence assay was performed on teased fibers of murine sciatic nerves. Specifically, teased fibers were dissected from the sciatic nerves of adult C57BL/6J mice on adhesion microscope slides and fixed in acetone at room temperature for 10 min. The slides were permeabilized with 1% Triton X-100 at 37°C for 30 min, blocked, and incubated with sera diluted at 1:10 with PBS together with chicken anti-human/mouse/rat neurofascin antigen affinity-purified polyclonal antibodies (1:50; R&D Systems, Minneapolis, MN, USA) overnight at 4°C, and then incubated with AffiniPure goat anti-human IgG Fcc (1:200; Alexa Fluor 594; Jackson ImmunoResearch) together with AffiniPure goat anti-chicken IgY (IgG) (H + L) (1:200; Alexa Fluor 488; Jackson ImmunoResearch) at 37°C for 45 min. Images were acquired using a fluorescence microscope (Leica).

### Neuroimaging, electrophysiological data, and sural nerve biopsy analyses

Magnetic resonance imaging (MRI) of the lumbosacral nerve roots was performed using a 3.0 T MR scanner. The electrophysiological characteristics involved motor and sensory conduction in the upper and lower extremities. Motor distal latency, motor conduction velocity (MCV), and amplitude were recorded bilaterally using standard protocols. We calculated the terminal latency index (TLI) following the method previously described by Katz et al. (15) as follows: TLI = distal distance/(proximal conduction velocity × distal latency).

Sural nerve biopsy was performed on one patient. The specimen was divided into two portions. The first portion was fixed in 2.5% glutaraldehyde for toluidine blue staining and electron microscopy analysis. In electron microscopy observations, we observed this portion in longitudinal sections at a final magnification of 60 000× to assess the morphology of the nodes of Ranvier and the paranodes. The second portion was frozen in isopentane, which was pre-cooled in liquid nitrogen, and stored at -80°C. Frozen nerve sections were stained with hematoxylin and eosin, MGT, and Congo red. For immunohistochemistry, the primary antibody used was an anti-CD3 mouse monoclonal antibody (clone LN10; Zhongshan Golden Bridge Biotechnology, Beijing, China). The density of myelinated fibers was evaluated in transverse sections using a light microscope.

### Statistical analysis

Qualitative variables were expressed as percentages and absolute frequencies, while quantitative features were reported as mean and standard deviation (SD) values. All analyses were performed using SPSS 22.0 (IBM, NY).

## Results

### Patients with anti-CNTN1 antibody

**Figure 1:**
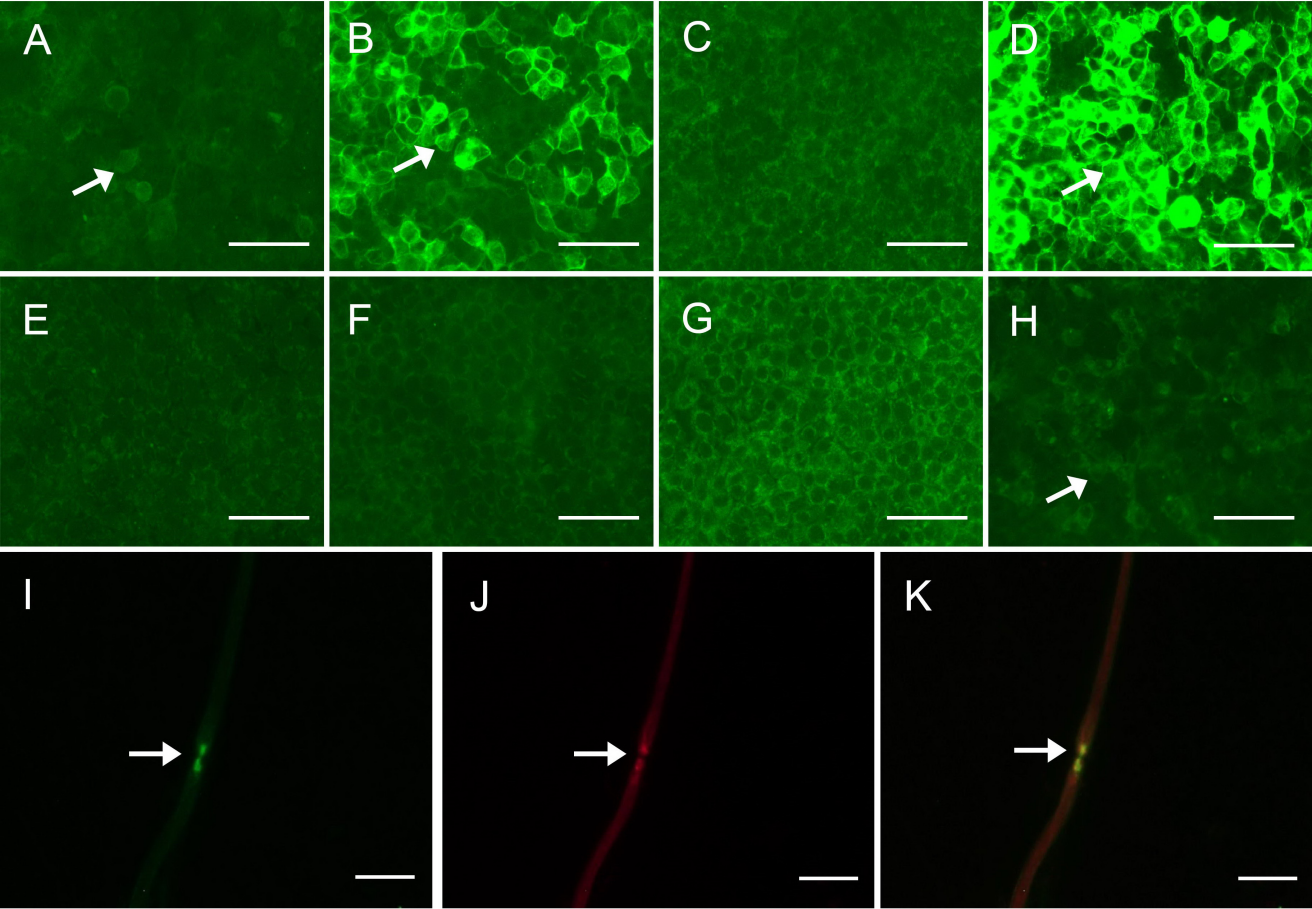
Detection of anti-CNTN1 antibodies **(A–D)** Detection of anti-CNTN1 antibodies in serum of patient 2. **(A)** 1:10 positive anti-CNTN1 IgG1 antibodies (arrow); **(B)** 1:320 positive anti-CNTN1 IgG2 antibodies (arrow); **(C)** Negative anti-CNTN1 IgG3 antibodies; **(D)** 1:3200 positive anti-CNTN1 IgG4 antibodies (arrow); **(E–H)** Detection of anti-CNTN1 antibodies in CSF of patient 1. **(E)** Negative anti-CNTN1 IgG1 antibodies; **(F)** Negative anti-CNTN1 IgG2 antibodies; **(G)** Negative anti-CNTN1 IgG3 antibodies; **(H)** 1:1 positive anti-CNTN1 IgG4 antibodies (arrow); **(I–K)** Double-immunofluorescence of murine teased fibers with serum sample of the patient. Seropositive of serum from patient 1 (green) (arrow). Antigen was in the paranodal region as the serum antibody merged well to HEK293 cells transfected with human NF155/NF186 plasmids (yellow) (arrow). Scale bars = 100 μm **(A–H)**; Scale bars = 50 μm **(I–K)**. CNTN1 = contactin-1; CSF, cerebrospinal fluid; NF155/NF186, neurofascin-155/neurofascin-186.

**Figure 2:**
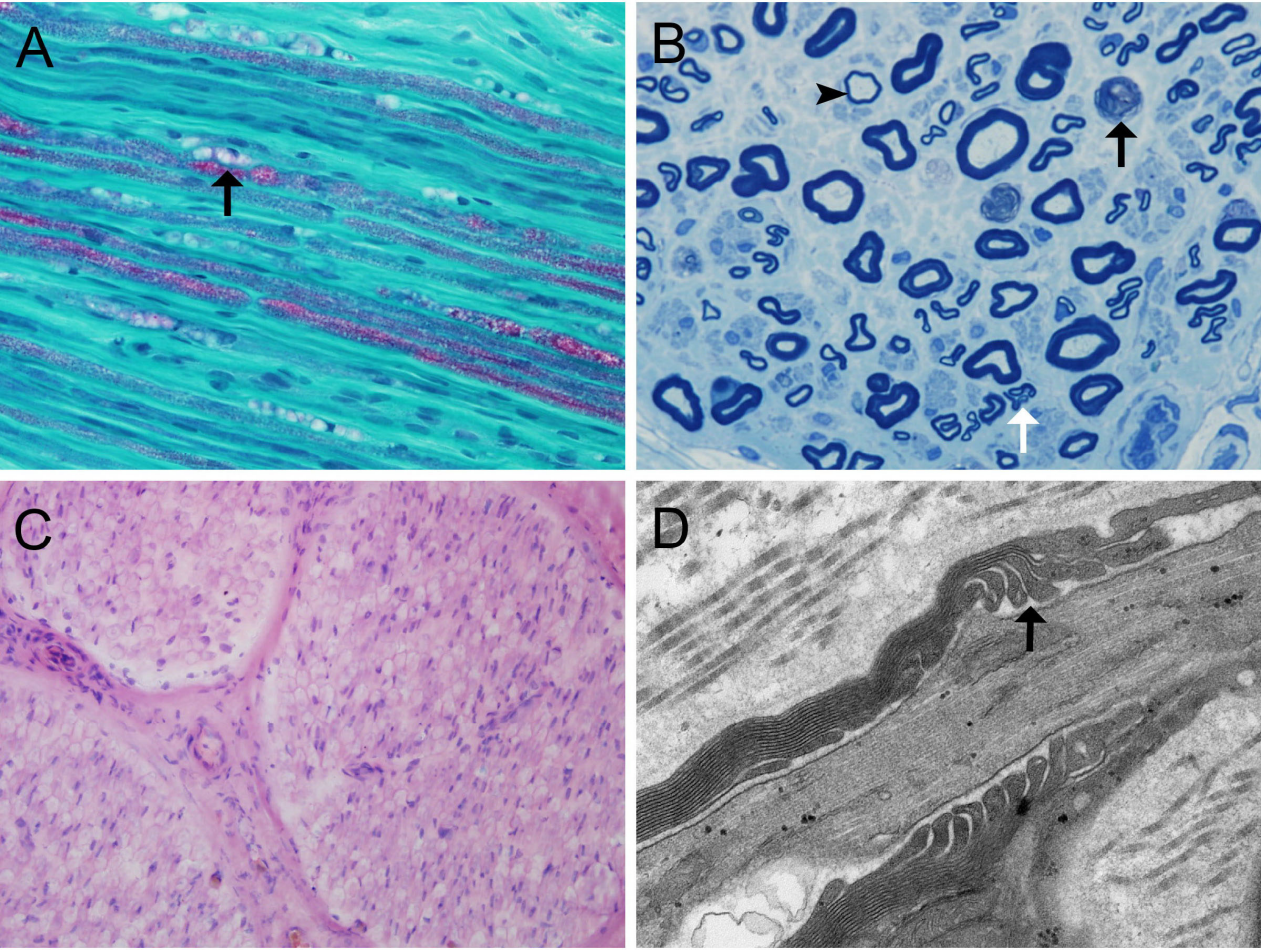
Nerve biopsy findings in anti-CNTN1-positive autoimmune nodopathy **(A)** MDC (arrow) and slightly reduced myelinated fibers in patient 1; **(B)** Myelin ovoids (black arrow), clusters of regenerating nerve fibers (white arrow), and thinly myelinated fiber (arrowhead) though semithin toluidin blue staining in patient 1; **(C)** No obvious subperineurial edema or lymphocytic infiltration in patient 1; **(D)** Large spaces observed between the terminal loops and the axolemma under electron microscopy (arrow) in patient 1. Scale bars = 50 μm (A, C); Scale bars = 10 μm **(B)**; Scale bars = 500 nm **(D).** CNTN1, contactin-1; MDC, myelin digestion chamber;

We identified 2 patients harboring antibodies against CNTN1 that met the inclusion criteria. One showed anti-CNTN1 IgG2 and anti-CNTN1 IgG4 positivity, and another showed anti-CNTN1 IgG1, anti-CNTN1 IgG2, and anti-CNTN1 IgG4 positivity (**Figure 1**). Both of these 2 anti-CNTN1 positive patients were male. The age at enrollment was 52.00 ± 2.83 years. Indeed, they all showed resistance to IVIg and prednisone when they visited our clinic. Specifically, 6 months before patient 1 came to our hospital, he received IVIg (0.4 g/kg/day for 5 days), and intravenous methylprednisolone (1 g/day for 5 days) followed by oral prednisolone (60 mg/day). And prednisolone was gradually tapered. Clinical features including unsteady gait, muscle weakness, dysphagia, numbness and tremor were not improved, thus intravenous cyclophosphamide (0.6 g q2w) was added as adjunctive treatment. Although this cyclophosphamide regimen led to disappearance of dysphagia and slight reduction in muscle weakness, it was stopped after cumulated dose of 3.6 g since the treatment led to elevated levels of aspartate transaminase and alanine transaminase. In patient 2, IVIg (0.4 g/kg/day for 5 days) and intravenous methylprednisolone (1 g/day for 2 days and 0.5 g/day for 2 days) followed by oral prednisolone (65 mg/day) which gradually tapered as reducing the dose by 5mg every two weeks were admitted before he visited our hospital. Although slight improvement was obtained in field of unsteady gait and muscle weakness about 1 month after treatment, these manifestations got worse when prednisolone was reduced to 30mg. Both patients had muscle weakness, unsteady gait, numbness, tremor, high CSF protein, and diffuse thickening of the lumbosacral nerve roots when they admitted in our hospital (**Table 1**). Sural nerve biopsy showed a slight reduction in myelinated fiber density and scattered myelin digestion chamber without onion bulb formation and inflammatory infiltration in patient 1. Electron microscopy of the longitudinal sections revealed a clear space between the Schwann cell terminal loops and axolemma (**Figure 2**).

**Table 1:** Clinical and histopathological features of two patients with autoimmune nodopathy with anti-CNTN1 antibody.

Features	Patient 1	Patient 2
Demographics		
Age at onset (y)	50	54
Sex	male	male
Clinical features		
Duration from disease onset to examination in our hospital (m)	10	17
Cranial nerve involvement	+	–
Proximal muscle weakness	+	+
Distal muscle weakness	+	+
Sensory ataxia	+	+
Numbness	+	+
Tremor	+	+
Neuropathic pain	–	–
Hughes disability scale overall	3	3
mRS	4	3
ODSS in upper limbs	3	3
ODSS in lower limbs	4	4
Serum Anti-CNTN 1 antibody	IgG2 1:10, IgG4 1:100	IgG1 1:10, IgG2 1:320, IgG4 1:3200
CSF Anti-CNTN 1 antibody	IgG4 1:1	IgG4 1:10
Electrophysiological study		
Prolonged distal motor latency	+	+
Reduced motor conduction velocity	+	+
Conduction block	+	+
Renal involvement		
Urine protein	Strongly positive	Mild positive
IgGU (< 14 mg/L)	33.8	6.14
Anti-PLA2R antibody	–	–
CSF protein (g/L)	3.45	5.22
Nerve root thickening on MRI	+	+
Peripheral CD19+ B cell (%)	13.08	12.61
Nerve biopsy		
Space between the Schwann cell terminal loops and the axolemma	+	ND
Myelin digestion chamber	+	ND
Onion bulb formation	–	ND
Inflammatory infiltration	–	ND

CNTN1, contactin-1; CSF, cerebrospinal fluid; IgGU, IgG in urine; MRI, magnetic resonance imaging; mRS, modified Rankin score; ND, not done; ODSS, overall disability sum score; PLA2R, phospholipase A2 receptor; TLI, terminal latency index.

### Evaluation of low-dose rituximab for clinical features in patients with anti-CNTN1 antibodies

Both patients experienced improvements in neurological symptoms following rituximab treatment. Indeed, sensory ataxia improved rapidly only two days after low-dose rituximab treatment. Other clinical symptoms, such as muscle weakness, tremor, and numbness, improved within 2 weeks following low-dose rituximab treatment and showed steady progress (**Table 2**). However, nephrotic damage, such as urinary IgG and albumin, did not respond to the low-dose rituximab regimen (**Table 3**).

**Table 2:** Clinical features of two autoimmune patients with nodopathy with anti-CNTN1 antibody after low-dose rituximab treatment.

Features	2 days	14 days	6 months
Improvement of cranial nerve (%)	0 (0)	1 (100)	1 (100)
Improvement of proximal muscle weakness (%)	0 (0)	2 (100)	2 (100)
Improvement of distal muscle weakness (%)	0 (0)	2 (100)	2 (100)
Improvement of sensory ataxia (%)	2 (100)	2 (100)	2 (100)
Improvement of numbness (%)	0 (0)	2 (100)	2 (100)
Improvement of tremor (%)	0 (0)	2 (100)	2 (100)
Dose of prednisone usage (mg)	60 ± 0.00	60 ± 0.00	25 ± 7.07
-△Hughes	0.00 ± 0.00	1.00 ± 0.00	2.50 ± 0.71

CNTN1, contactin-1.

**Table 3:** Laboratory findings of two patients with anti-CNTN1 antibody before and after low-dose rituximab treatment.

Findings		Baseline		2 days		14 days		6 months	
Patient 1		Patient 2	Patient 1	Patient 2	Patient 1	Patient 2	Patient 1	Patient 2
Serum anti-CNTN1 antibody								
IgG	1:100	1:3200	1:32	1:3200	ND	1:1000	1:10	–
IgG 1	–	1:10	–	–	ND	–	–	–
IgG 2	1:10	1:320	1:10	1:100	ND	1:32	–	–
IgG 3	–	–	–	–	ND	–	–	–
IgG 4	1:100	1:3200	1:32	1:1000	ND	1:1000	1:10	–
Total IgG (700-1600 mg/dl)	981	834	ND	ND	856	819	ND	ND
IgGU (< 14 mg/L)	33.8	6.14	58.2	ND	ND	ND	39	23.1
AlbU (< 30 mg/L)	1710	25.3	3420	ND	ND	ND	2560	449
Peripheral CD19+ B cells	13.08	12.61	0.69	0.28	0.04	0.00	0.023	0.28

CNTN1, contactin-1; IgGU, IgG in urine; AlbU, albumin in urine; ND, not done.

The clinical function score △Hughes showed partial effectiveness of rituximab treatment after 2 days and sustained effectiveness after 14 days (**Table 2**). Concurrently, the dose of prednisolone in these two patients also gradually declined and was lowered to below 30 mg/day at 6 months (**Table 2**). No severe adverse events were observed during the 6-month follow-up period.

### Evaluation of low-dose rituximab for electromyography features in patients with anti-CNTN1 antibodies

Electrodiagnostic studies in both patients showed a reduction in MCV, prolongation of distal motor latency, and prolongation of F-wave latencies. In addition, amplitude reduction of the motor nerve was observed, and sensory nerve action potentials were absent. Indeed, marked improvements were found in electrophysiological fields including MCV, F-wave latencies, the amplitude of the motor nerve, and velocity and amplitude of sensory nerve conduction at 6 months after low-dose rituximab treatment in patient 1 (**Supplementary Table 1**).

### Evaluation of low-dose rituximab for anti-CNTN1 antibody and its subclasses in patients with anti-CNTN1 antibodies

In both patients, anti-CNTN1 IgG was detected in the serum and CSF using the cell-based assay method. We confirmed that the serum showed reactivity against the paranodal region by an immunofluorescence assay on teased fibers of murine sciatic nerves. Indeed, patient 1 had 1:10 positive anti-CNTN1 IgG2 and 1:100 positive anti-CNTN1 IgG4 in the serum, and 1:1 positive anti-CNTN1 IgG4 in the CSF. Patient 2 had 1:10 positive anti-CNTN1 IgG1, 1:320 positive anti-CNTN1 IgG2, 1:3200 positive anti-CNTN1 IgG4 in serum, and 1:10 positive anti-CNTN1 IgG4 in CSF (**Figure 1**).

The titer of the anti-CNTN1 antibody subclasses decreased rapidly and steadily in the serum. Specifically, except for the anti-CNTN1 IgG2 antibody in patient 1, the titer of other subclasses of anti-CNTN1 antibody in both patients decreased only two days following low-dose rituximab treatment. At the 6-month follow-up, anti-CNTN1 IgG2 antibodies were negative for anti-CNTN1 IgG4 antibodies down to 1:10 in patient 1, and all subclasses of anti-CNTN1 antibodies were negative in patient 2 (**Table 3**).

### Evaluation of low-dose rituximab for B cell depletion in patients with anti-CNTN1 antibodies

The proportion of CD19+ B cells in the lymphocytes was normal in both patients before rituximab administration. Indeed, it decreased sharply only 2 days following rituximab infusion, from 13.08% to 0.69% and 12.61% to 0.28% in two patients, respectively. This number was sustained below 1% even at a 6-month follow-up. The proportion of CD19+ B cells before and after low-dose rituximab treatment is shown in **Table 3**.

## Discussion

Our study is the first to report the use of low-dose rituximab in patients with anti-CNTN1 autoimmune nodopathy. We observed rapid and sustained clinical recovery, subclasses of anti-CNTN1 IgG decrease and B cells decrease without adverse events in our anti-CNTN1-positive patients who had poor response to the first-line immunotherapies. Thus, low-dose rituximab may have a rapid and prolonged response with safety in anti-CNTN1 autoimmune nodopathy.

Although antibodies targeting proteins at the node and paranode of Ranvier, such as NF155, CNTN1, Caspr1, NF186, and NF140, were first described in a small subgroup of patients with CIDP, the immunopathological mechanisms of patients with these antibodies were largely related to the disruption of the nodal or paranodal region structure, which was different from the peripheral nerve demyelination in typical CIDP (3). Therefore, patients with these antibodies are regarded as a separate entity called autoimmune nodopathies instead of a subgroup of CIDP in the recent update of the European Academy of Neurology/Peripheral Nerve Society CIDP diagnostic guidelines (1).

Antibodies directed against CNTN1 result in detachment of terminal myelin loops and mislocalization of juxtaparanodal potassium channels to the paranode (16). IgG4 is regarded as the main pathogenic IgG subtype of anti-CNTN1 antibodies. Similar to previous studies, our patients showed few responses to IVIg when they visited our clinic (9, 17). This may be because the major effector function of IVIg is complement binding through binding to various Fc receptors and complement activation, while IgG4 does not bind Fc receptors and does not activate the complement pathway (18). Therefore, alternative treatments, instead of traditional immunotherapies, are needed for anti-CNTN1 autoimmune nodopathy (19).

Rituximab is a monoclonal antibody against CD20, a B cell surface antigen present on pre-B cells and throughout the B cell life cycle. Circulating CD20+ B cells become undetectable almost immediately after the first dose of rituximab. Previous reports showed that IgG4 autoantibodies against MuSK and IgG4 autoantibodies against NF155 decrease rapidly after CD20+ B cell depletion with rituximab (7, 20, 21). Besides, decrease of antibody with clinical improvement was also seen in other IgG4-ND such as anti-LGI1 encephalitis, CASPR2-associated syndromes and in IgG4-RD (10, 22–27). As most of the antibody-secreting cells (ASCs) are CD20- and are not directly targeted by rituximab, titer reduction was explained by depletion of the CD20+ ASC-progenitor cells in combination with the short-lived nature of ASCs (28–30). We presume that a similar mechanism exists in our patients. The rapid and sustained response to rituximab suggests that CNTN1 antibodies are likely produced by short-lived antibody-secreting cells, a cell pool that needs to be constantly refilled from the B cell compartment. Therefore, the treatment of rituximab could lead to decrease of antibody titer in a short time. Other mechanism of titer reduction may also exist, and further exploration is needed. Since anti-CNTN1 antibodies may have an antigen-blocking function by blocking the interaction between CNTN1 and its partners CNTNAP1/Caspr1 at the paranode (31), rituximab may have a positive effect by reducing the generation of anti-CNTN1 autoantibodies. In fact, patients with autoimmune diseases generally have long-lasting and profound outcomes after rituximab treatment, including dramatic clinical improvement, longer time to the next relapse, and decreased post-treatment antibody titers, especially for those who are refractory to IVIg or prednisone (5, 32–34). In anti-CNTN1 autoimmune nodopathy, most patients showed stabilized improvement and autoantibody titers decreased without adverse events even after one year of rituximab treatment (7–9), while 2 patients shortly died after the first rituximab injection (5, 35).

The most common rituximab regimen was an intravenous administration of 375 mg/m^2^/week over 4 weeks. Since rituximab is not covered by social security in China, the high cost of treatment largely limits its administration (36). We analyzed two patients with anti-CNTN1 antibodies treated with low-dose rituximab over six months. We found that one cycle of low-dose rituximab led to rapid and sustained clinical improvement in patients with anti-CNTN1 antibodies. Specifically, unsteady gait improved only two days after rituximab treatment. All clinical neurological manifestations including muscle weakness, tremor and numbness significantly improved at 14 days, and steroid doses were reduced to below 30 mg at 6 months. Indeed, low-dose rituximab exerts its effect through B cell elimination in our anti-CNTN1-positive patients since CD19+ B cells quickly decreased to less than 1% and remained within 6 months after infusion. Our case study suggests that the low-dose regimen results in a rapid and profound response to anti-CNTN1 autoimmune nodopathy. This favorable outcome may be due to the relatively short disease duration, as early treatment is significant to avoid permanent nerve damage (7). Moreover, the rapid improvement of sensory ataxia to rituximab in our patients may share similar reason to reversible conduction failure (RCF) in CIDP. It has been reported of rapid recovery of CIDP, which is difficult to explain by the process of remyelination (37). In fact, disease is arrested with a rapid reappearance and reorganization of voltage-gated sodium channel at the nodal region leading to RCF before the development of axonal degeneration. We assumed that antibodies binding to CNTN1 disrupt voltage-gated ion channels and axon-glial junction at the paranodes may be eventually arrested after rituximab therapy. More research is needed to further reveal the mechanism.

However, no obvious improvement in the patient’s nephrotic features was observed. In fact, patients with anti-CNTN1 autoimmune nodopathy often had renal involvement with membranous nephropathy as granular deposits of IgG4 along the glomerular basement membrane in renal biopsies and the weakly expression of CNTN1 in the kidney (5, 35, 38, 39). Both of our patients demonstrate glomerular injury, the renal dysfunction may result from membranous nephropathy. However, rituximab was only used in a few anti-CNTN1-positive nodopathy patients with membranous nephropathy. Specifically, 2 patients shortly died after the first rituximab injection (5, 35). Another 3 patients treated with rituximab of 650mg once, 375 mg/m^2^ weekly for 4 consecutive weeks and of 375 mg/m^2^ twice respectively showed remission of nephrotic syndrome (40–42). Standard-dose of rituximab was recommended in membranous nephropathy and only 60–70% of patients could reach persistent clinical remission (43), the same protocol has been used to treat immune related peripheral neuropathy. Recently, researchers found that autoimmune nodopathy patient with low-dose of rituximab could also receive favorable outcomes, while the effect of low-dose rituximab regimen in membranous nephropathy was controversial (44, 45). The difference effect about low-dose rituximab between autoimmune nodopathy and membranous nephropathy, and the treatment regimen for nephrotic involvement in patients with anti-CNTN1 antibody need to be further explored. Importantly, neither of our two patients suffered from the side effects of our low-dose rituximab regimen, suggesting that this regimen would be safe for anti-CNTN1 nodopathy.

Furthermore, previous reports have shown that patients with autoimmune diseases, especially in the Chinese cohort, including anti-NF155 autoimmune nodopathies, MG, NMOSD, and Isaacs syndrome, responded very well to the low-dose rituximab regimen (10, 46–49). This low-dose regimen could also lead to improvement of clinical manifestations, such as manual muscle testing and disease-related scales, decrease in antibody titers, and reduction of average steroid dosage by B cell elimination and subsequent B cell repopulation. Our finding that patients with anti-CNTN1 antibodies had a favorable response to low-dose rituximab treatment could extend the off-label use of a low-dose rituximab regimen. We suggest that low-dose rituximab is an effective and safe treatment regimen for autoimmune diseases in China.

Autoimmune nodopathy with anti-CNTN1 is a rare disease. The small number of patients with anti-CNTN1 nodopathy is the main limitation of our study. Thus, the rapid and profound response to the low-dose rituximab regimen needs to be confirmed in further studies, especially in other ethnic groups. Besides, the total amount of IgG4 level were not performed in both patients. More attention on this point needs to be paid in the further study.

In conclusion, this small, self-controlled pilot study showed the profound efficacy of single low-dose rituximab in clinical symptom improvement, anti-CNTN1 antibody decrease, and steroid dose reduction in anti-CNTN1 nodopathy through B cell elimination. Since the response to this low-dose rituximab regimen is long-lasting and safe, we suggest that low-dose rituximab should be used early in anti-CNTN1 nodopathy. Given that our present study contained only a relatively small sample of patients with anti-CNTN1 nodopathy, future studies based on larger cohorts of patients are required to support our conclusions.

## Data availability statement

The original contributions presented in the study are included in the article/**Supplementary Material**. Further inquiries can be directed to the corresponding author.

## Ethics statement

The studies involving human participants were reviewed and approved by the ethics committee of Qilu hospital. The patients/participants provided their written informed consent to participate in this study.

## Author contributions

Study concepts, patient evaluation, data acquisition, interpretation and analysis, and manuscript drafting: YH. Study design and data acquisition: XY, DZ, CY, LM, JJ, AL, and LC. Anti-CNTN1 testing: WW. Manuscript revision: CZ and YB. Study design, interpretation of results, and manuscript revision: QW. All authors contributed to the article and approved the submitted version.

## Funding

This study was supported by the National Natural Science Foundation of China (Grant No. 82071412), Natural Science Foundation of Shandong Province (Grant No. ZR2021MH337 and ZR2021QH120), and Key Research and Development Project of Shandong Province (2019GSF108064).

## Conflict of interest

The authors declare that the research was conducted in the absence of any commercial or financial relationships that could be construed as potential conflict of interest.

## Publisher’s note

All claims expressed in this article are solely those of the authors and do not necessarily represent those of their affiliated organizations, or those of the publisher, the editors and the reviewers. Any product that may be evaluated in this article, or claim that may be made by its manufacturer, is not guaranteed or endorsed by the publisher.
