# Supplementary material for: Effect of low-dose rituximab treatment on autoimmune nodopathy with anti-contactin 1 antibody
Source: Front Immunol. 2022 Jul 26;13:939062. doi: 10.3389/fimmu.2022.939062 (PMC9362773; doi:10.3389/fimmu.2022.939062)
Supplement: Supplementary file 1 [file Table_1.docx]

Supplement Table 1 Detailed electrophysiologic data of patient 1 before and after low-dose rituximab regimen

|  | Before rituximab treatment | | | After 6-month rituximab treatment | | |
| --- | --- | --- | --- | --- | --- | --- |
|  | Left | | Right | Left | | Right |
| MOTOR |  |  | |  |  | |
| Ulnar nerve |  |  | |  |  | |
| DML, ms | 6.54 | 6.54 | | 3.29 | 3.17 | |
| CV, m/s | 22.9 | 28.2 | | 46.1 | 40.7 | |
| CMAP amplitude | 2.3 | 2.3 | | 3.9 | 3.0 | |
| TLI | 0.33 | 0.27 | | 0.33 | 0.39 | |
| Median nerve |  |  | |  |  | |
| DML, ms | 7.88 | 8.46 | | 3.98 | 4.25 | |
| CV, m/s | 28.2 | 24.9 | | 40.5 | 40 | |
| CMAP amplitude | 2.2 | 3.3 | | 2.8 | 3.7 | |
| TLI | 0.27 | 0.28 | | 0.37 | 0.35 | |
| Tibial nerve |  |  | |  |  | |
| DML, ms | NR | NR | | NR | NR | |
| CV, m/s | NR | NR | | NR | NR | |
| CMAP amplitude | NR | NR | | NR | NR | |
| TLI | NA | NA | | NA | NA | |
| Peroneal nerve |  |  | |  |  | |
| DML, ms | NR | NR | | NR | NR | |
| CV, m/s | NR | NR | | NR | NR | |
| CMAP amplitude | NR | NR | | NR | NR | |
| TLI | NA | NA | | NA | NA | |
| SENSORY |  |  | |  |  | |
| Ulnar nerve |  |  | |  |  | |
| SNAP | NR | NR | | 6.0 | ND | |
| CV | NR | NR | | 44.3 | ND | |
| Median nerve |  |  | |  |  | |
| SNAP | NR | NR | | 11.7 | 7.2 | |
| CV, m/s | NR | NR | | 51.4 | 53.3 | |
| Superficial peroneal nerve |  |  | |  |  | |
| SNAP | NR | NR | | 3.6 | 3.9 | |
| CV, m/s | NR | NR | | 35.6 | 35.5 | |

Abbreviations: DML, distal motor latency; CV, conduction velocity; CMAP, compound muscle action potential; TLI, terminal latency index; SNAP, sensory nerves action potentials; NA, not applicable; ND, not done; NR, No response.
