# Supplementary material for: Effect of low-dose rituximab treatment on autoimmune nodopathy with anti-contactin 1 antibody
Source: Front Immunol. 2022 Jul 26;13:939062. doi: 10.3389/fimmu.2022.939062 (PMC9362773; doi:10.3389/fimmu.2022.939062)
Supplement: Supplementary file 2 [file Table_2.docx]

Supplement Table 2 Detailed electrophysiologic data of patient 2 before and after low-dose rituximab regimen

|  | Before rituximab treatment | | | After 6-month rituximab treatment | | |
| --- | --- | --- | --- | --- | --- | --- |
|  | Left | | Right | Left | | Right |
| MOTOR |  |  | |  |  | |
| Ulnar nerve |  |  | |  |  | |
| DML, ms | 6.88 | 4.81 | | 4.67 | 3.48 | |
| CV, m/s | 44.5 | 52 | | 51.7 | 52.3 | |
| CMAP amplitude | 2.6 | 4.1 | | 3.4 | 4.4 | |
| TLI | 0.16 | 0.20 | | 0.20 | 0.27 | |
| Median nerve |  |  | |  |  | |
| DML, ms | 8.71 | 10.9 | | 4.82 | 4.91 | |
| CV, m/s | 36.6 | 38.5 | | 51.2 | 49.8 | |
| CMAP amplitude | 2.4 | 4.2 | | 4.2 | 5.0 | |
| TLI | 0.19 | 0.14 | | 0.24 | 0.25 | |
| Tibial nerve |  |  | |  |  | |
| DML, ms | 13.2 | 11.1 | | 6.47 | 5.98 | |
| CV, m/s | 27.5 | 26.0 | | 36.7 | 36.5 | |
| CMAP amplitude | 0.18 | 0.17 | | 0.46 | 0.37 | |
| TLI | 0.28 | 0.35 | | 0.42 | 0.46 | |
| Peroneal nerve |  |  | |  |  | |
| DML, ms | 9.89 | 9.28 | | 5.39 | 5.07 | |
| CV, m/s | 20.5 | 24.6 | | 31.4 | 33.3 | |
| CMAP amplitude | 0.28 | 0.32 | | 0.65 | 1.02 | |
| TLI | 0.39 | 0.35 | | 0.47 | 0.47 | |
| SENSORY |  |  | |  |  | |
| Ulnar nerve |  |  | |  |  | |
| SNAP | NR | NR | | 3.8 | 2.2 | |
| CV | NR | NR | | 41.3 | 42.5 | |
| Median nerve |  |  | |  |  | |
| SNAP | NR | NR | | 4.6 | 8.2 | |
| CV, m/s | NR | NR | | 48.9 | 51.2 | |
| Superficial peroneal nerve |  |  | |  |  | |
| SNAP | 6.6 | 2.9 | | 9.6 | 5.0 | |
| CV, m/s | 39.4 | 41.2 | | 47.6 | 45.5 | |

Abbreviations: DML, distal motor latency; CV, conduction velocity; CMAP, compound muscle action potential; TLI, terminal latency index; SNAP, sensory nerves action potentials; NR, No response.
